# Episodic Gregariousness Leads to Level‐Dependent Core Habitats: A Case Study in Eastern Copperheads (*Agkistrodon contortrix*)

**DOI:** 10.1002/ece3.70788

**Published:** 2025-01-08

**Authors:** Tyler C. Christensen, Robert Kwait, Michael Van Clef, Brooke Maslo

**Affiliations:** ^1^ Department of Ecology Evolution & Natural Resources Rutgers University New Brunswick New Jersey USA; ^2^ Friends of Hopewell Valley Open Space Pennington New Jersey USA

**Keywords:** habitat selection, habitat use, kernel density estimation, multilevel selection, radio telemetry, resource utilization functions

## Abstract

Characterizing the complex relationships between animals and their habitats is essential for effective wildlife conservation and management. Wildlife–habitat selection is influenced by multiple life‐history requirements, which act over varying spatial and temporal scales, and result in dispersion patterns that can differ across ecological levels. For example, sites that attract intense communal use (e.g., hibernacula and communal basking sites) are often a subset of the habitats required by individuals for survival. Despite the conservation importance of both individually and communally significant habitats, snake habitat models rarely incorporate information about both individual and population‐level activity. We used 4 years of radiotelemetry data from eastern copperheads ( *Agkistrodon contortrix* ) to evaluate the presence of multilevel spatial habitat responses and whether they revealed conservation‐relevant information. We related individual and population space use intensity to underlying habitat covariates to determine whether predictors of copperhead spatial activity were level‐dependent, and whether individual core habitats differed by sex and reproductive state. Copperheads' episodic gregariousness resulted in spatial and environmental separation between individual and communal core habitats. Population‐level use was greatest in rocky, forested habitats associated with winter brumation and spring basking, whereas individual‐level use was greatest in open habitats with woody debris associated with foraging and reproductive behaviors. Male core habitats were open and thickly vegetated while those of females were moderately forested, with gravid female core habitats containing ample woody debris. Our findings demonstrate that multilevel spatial patterns carry conservation‐relevant information about snake habitat relationships. We suspect that behaviors leading to multilevel spatial patterns exist in many wildlife species whose individual spatial activities overlap around shared resources.

## Graphical abstract

Episodic gregariousness resulted in separation of individual and population core habitats in eastern copperheads ( *Agkistrodon contortrix* ). Core habitats of males, gravid females, and nongravid females also differed significantly. Investigating multiple levels of spatial activity revealed conservation‐relevant information about snake habitat relationships.

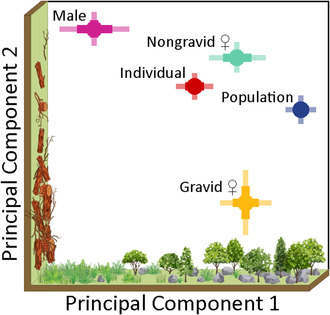

## 1 Introduction

Identifying relationships between animals and their resources is essential for effective wildlife management and conservation. Wildlife–resource interactions are inherently complex; yet, understanding them provides fundamental information about how animals meet their fitness requirements. A particular challenge facing wildlife biologists is that the strength and direction of wildlife resource selection can vary across spatial and temporal scales (Johnson 1980; Boyce 2006; McGarigal et al. 2016), as well as ecological levels (e.g., individuals, groups, subpopulations, populations, etc.; Johnson 1980; DeCesare et al. 2012; Johnson et al. 2016). Confining investigations to a single scale or level can result in misleading inferences regarding critical resources (Mayor et al. 2009; Bauder et al. 2018; Moraga et al. 2019) and missed opportunities for successful habitat management or protections. Therefore, ecologists have come to recognize that studies representing the multilevel nature of wildlife–resource interactions provide more accurate characterizations of resource needs (Zeller et al. 2017; Bauder et al. 2018).

Habitat selection is a process whereby individual animals use habitats disproportionately more or less than their availability (Johnson 1980; Matthiopoulos et al. 2023). Because resources are rarely homogeneously distributed, selection typically results in spatially heterogeneous habitat use (Marzluff et al. 2004; Matthiopoulos et al. 2023). Spatial use variation can be modeled with the utilization distribution (UD), a continuous three‐dimensional surface estimating the probability density of an animal being found at a given point in geographic space (Marzluff et al. 2004; Millspaugh et al. 2006). The UD can be regressed against spatial predictors (i.e., resources) to fit a resource utilization function (RUF) and obtain inferences about the composition and spatial distribution of intensely used habitats (Marzluff et al. 2004; Millspaugh et al. 2006). Because habitat use is a direct consequence of habitat selection, RUFs are closely related to resource selection functions (RSFs), and their parameters converge under specific sampling conditions (Hooten et al. 2013; Matthiopoulos et al. 2023).

**FIGURE 1:**
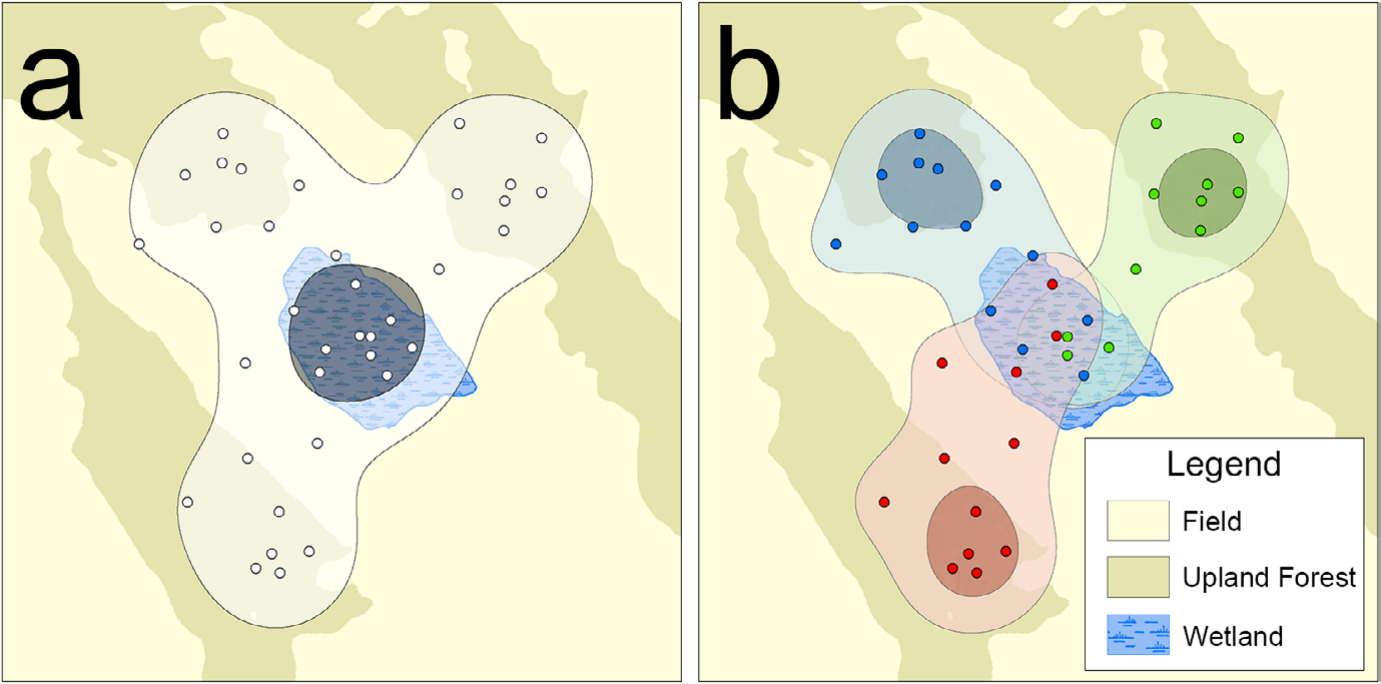
Spatially and environmentally distinct core habitats identified by population (a) and individual (b) utilization distributions (UDs). The shapes of the UDs are based on the hypothetical movements of three individual animals. Population‐level core habitat is a shared wetland, while the same observations differentiated by individual reveal individual core habitat as upland forest.

While multilevel study designs provide more complete inferences about wildlife–resource relationships, they typically do not consider multilevel spatial patterns in animal movements. Habitats that draw high levels of individual use (i.e., individual core areas) can be geographically and environmentally distinct from habitats with overlapping use by many individuals (i.e., population core areas; Johnson et al. 2016; Buechley et al. 2018). Such a phenomenon could arise from episodic gregariousness (e.g., reproduction, hibernation, or socialization) or from idiosyncratic individual selection driven by unknown or unidentified resources. In any case, areas with overlapping individual‐level use can result in intense population‐level use (Buechley et al. 2018; Winner et al. 2018). Population core areas could arise in communally used habitats (e.g., waterholes, travel corridors, breeding wetlands, etc.) even when constituent individuals concentrate their activity elsewhere (e.g., core areas of individual home ranges; Figure 1). Lack of consideration for multilevel spatial patterns could result in failure to detect habitats where disproportionate use occurs only at one level or another. This limitation has obvious conservation planning implications for species with complex habitat needs.

Reptiles, particularly snakes, exhibit complex habitat selection and spatial behaviors that are driven by their need to thermoregulate, avoid predators, and acquire food (Huey 1991; Reinert 1993; Pringle, Webb, and Shine 2003; Herr et al. 2020; Sutton et al. 2017; Bauder et al. 2018). In temperate latitudes, habitat selection often varies due to state‐specific physiological demands and changing environmental conditions, which can result in selection of distinctly different habitats needed to meet life‐history requirements (Reinert 1984; Waldron, Lanham, and Bennett 2006; Harvey and Weatherhead 2006; Gregory and Tuttle 2016). Structural resources, such as rock cover, canopy density, and ground layer vegetation, directly influence thermoregulatory opportunities by altering light intensity, air and ground temperatures, ambient moisture, and wind speed (Reinert 1993; Vitt, Zani, and Lima 1997; Litzgus and Brooks 2000; Row and Blouin‐Demers 2006). Many temperate‐latitude snakes overwinter communally in underground hibernacula, sometimes in mixed‐species assemblages (Woodbury 1951; Carpenter 1953; Parker and Brown 1973; Brown and Parker 1976). Species and populations exhibit varying degrees of hibernaculum fidelity, but usually individuals return to the same site in successive years (Brown and Parker 1976; Harvey and Weatherhead 2006). Suitable hibernacula become increasingly scarce, and therefore more communal, in progressively colder climates (Harvey and Weatherhead 2006). Generally, the aggregations that form at winter hibernacula dissolve when individuals disperse to summer habitats in spring (Gardiner et al. 2013; Bauder, Akenson, and Peterson 2015). Summer habitats used for foraging or reproduction can differ considerably from those used for hibernation (Reinert and Zappalorti 1988; Smith et al. 2009; Waldron, Lanham, and Bennett 2006; Gardiner et al. 2013). Consequently, there is a recurring alternation between gregarious and individualistic spatial behaviors (i.e., “episodic gregariousness”). The presence of such temporally, spatially, and intraspecifically variable habitat selection in snakes makes the existence of multilevel patterns of habitat use seem likely, though this has not been explicitly investigated.

Eastern copperheads ( *Agkistrodon contortrix* ) are a widespread pit viper (Crotalinae) found throughout much of eastern North America. Past studies on copperhead–habitat associations have either investigated individual‐level habitat selection (e.g., Reinert 1984, Sutton et al. 2017) or habitat use at the population level (Cross and Petersen 2001). Copperheads are episodically gregarious, forming seasonal aggregations (e.g., communal brumation and gestation; Fitch 1960; Graves and Duvall 1995), and undergoing annual migrations to and from foraging sites (Smith et al. 2009). They also exhibit variation in resource use linked to sex and reproductive status (Reinert 1984; Sutton et al. 2017). Therefore, they are ideal for investigating patterns of spatial resource use across multiple biological levels.

Here we used radiotelemetry data from four of monitoring eastern copperheads to determine whether: (1) patterns of spatial habitat use differed at the individual and population levels; and (2) individual high‐use (core) habitats varied by sex and reproductive status. We hypothesized that episodic gregariousness and intraspecific variation in copperhead habitat selection would result in spatial patterns of habitat use that were different for individuals and the population. We further hypothesized that incorporating these multilevel patterns into habitat use models explicitly would provide inferences that were biologically meaningful with respect to snake resource needs.

## 2 Materials and Methods

### 2.1 Study Area

We conducted this study in the Sourlands of New Jersey, an approximately 200‐km^2^ diabase sill situated in the northern piedmont plateau in the northeastern United States. The study area spanned private and preserved lands and was comprised of forest, field, and residential areas (buildings, roads, and lawns).

### 2.2 Snake Telemetry

We initially located copperheads during haphazard surveys of apparently suitable hibernation and basking habitats in spring. We captured copperheads using snake hooks and temporarily held them in ventilated lidded buckets for transportation. We then transported them to the Mercer County Wildlife Center (Titusville, New Jersey, USA) where they were housed for up to 48 h in well‐ventilated individual containers at room temperature. Individuals were not fed while in captivity but were given water ad libidum. We measured and implanted each snake with uniquely coded passive integrated transponder tags (AVID, Norco, California, USA) for permanent individual identification. We then surgically implanted adult snakes weighing greater than 190 g with radiotransmitters following the methods of Reinert and Cundall (1982) and Bryant et al. (2010). During anesthetization we manually restrained the copperheads within an acrylic snake tube. We induced anesthesia using a precision isoflurane vaporizer (Vapomatic 2, A.M. Bickford, Whales, New York, USA) which delivered a 5% isoflurane concentration in oxygen at 2 L/min. Once induced, we maintained anesthesia at 1.5% isoflurane. We then implanted transmitters (Model R1680, Advanced Telemetry Systems, Isanti, Minnesota, USA) into the rear third of the coelomic cavity. We implanted the antenna subcutaneously and oriented cranially using a 2‐mm‐diameter stainless steel catheter. We closed incisions with an over‐under interrupted stitch using absorbable sutures (4‐0 violet braided polyglycolic acid, Oasis, Mettawa, Illinois, USA). All surgical tools were either removed from sterile packaging or were sterilized using an autoclave prior to the procedure. To mitigate risks of postoperative inflammation and infection, we administered a nonsteroidal anti‐inflammatory drug (Metacam [meloxicam] 0.2 mg/kg; Boehringer Ingelheim, Macquarie Centre, New South Whales, Australia) and an antibiotic (ceftazidime 20 mg/kg; WG Critical Care, Paramus, New Jersey, USA) intraoperatively. T. Christensen performed all surgeries and administered all medications. While snakes were anesthetized, we determined sex by inserting a stainless‐steel probe into the cloaca to determine whether hemipenes were present. We determined whether females were gravid in summer when their abdomens began to enlarge with developing embryos and they began to display prolonged sedentary behavior. Presumed gravidity was confirmed in late summer when we observed females or their neonates following parturition. We released snakes to the point of capture within 48 h of surgery and relocated them every 48–72 h between 0800 and 2000 throughout the active season (early April to late October) using handheld receivers (Model R‐1000, Communications Specialists, Orange, California, USA, or IC‐R10, Icom, Kirkland, Washington, USA) connected to 3‐element folding Yagi antennas. All work was conducted under New Jersey Division of Fish & Wildlife Endangered and Nongame Species Program scientific collecting permit #SC2019022 and Rutgers University Institutional Animal Care and Use Committee protocol #202100005.

### 2.3 Field Data

Once a snake was relocated, we used a handheld GPS (GPSmap60csx, Garmin) to record its position in decimal degrees. At each snake relocation, we quantified five habitat variables within 1‐m^2^ quadrats centered on the snake's position, including percent cover of exposed surface rock, coarse woody debris (> 1 cm diameter), ground layer vegetation (< 0.5 m in height), woody understory vegetation (< 3.0 m in height), and percent canopy cover. The importance of these habitat features has been substantiated in previous studies of snake habitat use and selection (Reinert 1984; Reinert and Zappalorti 1988; Cross and Petersen 2001; Pringle, Webb, and Shine 2003; Sutton et al. 2017, etc.). We estimated canopy cover from nonhemispherical zenithal photographs taken on a smartphone and analyzed using binary thresholding in ImageJ (Macfarlane, Grigg, and Evangelista 2007; Chianucci 2015).

### 2.4 Data Analysis

#### 2.4.1 Home Ranges and Space Use

We estimated seasonal home ranges for each individual copperhead using 95% minimum convex polygons (MCP; Worton 1995). While the MCP approach is more sensitive to sample size and can include large areas with no known use (Burgman and Fox 2003; Crane et al. 2021), some authors recommend MCP over kernel‐based and probabilistic estimators for reptiles because it can produce more consistent home ranges (Row and Blouin‐Demers 2006). Additionally, because of its widespread use in the herpetological literature, the MCP approach maximizes comparability across studies. We visually assessed the effect of sample size on MCP home range estimates by incrementally subsampling each snake season, generating 95% MCPs from each subsample, and plotting the resulting areas as a function of sample size. MCP area stabilized with ≥ 20 observations (Figure S1). We therefore removed snake seasons that contained fewer than 20 observations and those that were incomplete due to mortality to improve accuracy of home range estimates and spatial activity models. We calculated MCP home ranges with the adehbitatHR package (Calenge 2006) using the statistical computing software R (R Core Team 2023).

We wished to investigate whether the habitat attributes predicting space use patterns were different at the individual and population levels. We modeled space use with utilization distributions (UDs), which provide important insight into the highly heterogeneous space use typical of reptiles caused by frequent or prolonged use of specific habitat features within the home range (e.g., sites used for foraging, basking, or reproduction; Row and Blouin‐Demers 2006; Silva et al. 2020). We estimated each individual UD and the population UD with kernel density estimation (KDE) using the MCP‐based bandwidth selection procedure recommended by Row and Blouin‐Demers (2006). Following this approach, we tuned the bandwidth for each animal or population such that the KDE‐UD was equal in size to the MCP. We first calculated the 95% MCP area and then iteratively generated kernel density UDs, increasing the bandwidth by 1‐m increments until the 95% volume contour (isopleth) of the UD equaled the area of the 95% MCP. For our data, this method consistently produced UDs with desirable characteristics, including multiple peaks per UD (i.e., not oversmoothing the UD), and those peaks tended to align with the dimensions and positions of important and intensely used snake habitats as we perceived them. We calculated MCP and KDE activity ranges using the adehbitatHR package (Calenge 2006) in R (R Core Team 2023).

#### 2.4.2 Multilevel Habitat Use

We performed the area‐optimized UD estimation described above to produce UDs at both the individual and population levels. At the individual level, we obtained one UD for each complete snake season. At the population level, we represented the spatial use of all individuals while accounting for variability in individual tracking durations (i.e., single‐ vs. multi‐season individuals) to control for the potential of multiseason individuals to disproportionately bias the UD. We controlled for this possible bias through a repeated random sampling procedure, where on each sampling iteration we randomly selected one snake season per individual, pooled observations across the set of randomly selected snake seasons, and generated one population‐level UD. The pooling of data across individuals to obtain a population‐level UD is analogous to previous approaches that inferred habitat use from population‐level data (Cross and Petersen 2001; Payer and Harrison 2003; Rittenhouse et al. 2008; Pearse et al. 2015). We repeated this procedure for 1000 iterations and averaged the resulting UDs using the package raster (Hijmans 2023) in R (R Core Team 2023).

We assigned two UD‐derived use intensity values (i.e., core and noncore) to each observation: one based on its position within its respective individual‐level UD, and one based on its position within the population‐level UD. We defined high‐use (i.e., core) areas as those within the 30% UD volume contour, and low‐use (i.e., noncore) areas as those outside the 30% volume contour to reduce misclassification of locations at intermediate positions within the UD as core or noncore habitat (see Donovan et al. 2011). Each tracked snake location was therefore associated with one set of measured habitat covariates and two use intensity values: one extracted from the individual‐level UD and one from the population‐level UD. We then used generalized linear models (glm) and generalized linear mixed models (glmm) to perform logistic regression with logit‐link functions using habitat covariates as predictors and the binary UD‐derived use intensity as the response. We applied a random effect of snake season to the population‐level model to control for individual variation in habitat use and reduce potential impacts caused by repeated sampling of individuals. Because we derived use levels in individual‐level models independently for each snake season, including a random effect of snake or snake season would not have been meaningful.

We initially attempted to address autocorrelation in utilization distributions by using autocorrelated kernel density estimation (Fleming et al. 2015) and dynamic Brownian bridge movement models (Kranstauber et al. 2012), but both techniques resulted in oversmoothed UDs that did not reflect fine‐scale spatial use intensity: in both approaches, clusters of observations associated with habitats of known importance either failed to produce probability density peaks in the UD or yielded broad, diffuse peaks that assigned unrepresentatively high‐use values to large areas of less used habitat surrounding the clusters. In contrast, the traditional KDEs appropriately assigned high‐use values to location clusters and low‐use values to less frequently used surrounding habitats, which more accurately reflected fine‐scale space use.

We generated semivariograms to confirm the presence of spatial autocorrelation in our habitat covariates (Figure S2) and compared GLMs and GLMMs with and without spatial random effects (Marzluff et al. 2004; Hooten et al. 2013). When unaddressed, spatial autocorrelation primarily results in underestimated confidence intervals for regression coefficients (Legendre et al. 2002). When we included spatial random effects, our models indicated no influence of habitat on copperhead movements (Supporting Information, Table A.1), contrary to prior studies (e.g., Reinert 1984; Cross and Petersen 2001; Sutton et al. 2017) and our field observations of substantial contrasts between the most and least used habitats. This may reflect spatial confounding bias (Paciorek 2010) which can occur when covariates and residuals share similar scales, such as when animal movements are highly correlated with habitat (Mercker et al. 2021). Some reptiles may be prone to such bias due to their tendency to exhibit highly concentrated and extended use of a small number of preferred habitats (Row and Blouin‐Demers 2006; Montano et al. 2022). We therefore report results from models without spatial random effects, with full results available in the Supporting Information (Table A.1).

To characterize population and individual‐level habitat use, we considered every possible combination of predictor covariates and calculated full model averages across candidate models that were < 2 ΔAIC from the top‐ranked model (Anderson and Burnham 2002). Each covariate was z‐score standardized so that effect sizes could be compared within and across models. All covariates were reasonably noncollinear (variance inflation factor [VIF] < 2). We considered the model‐averaged coefficients to be the relative effect sizes of the covariates on the log‐odds that an observation arose from a core area. We tested for significance in the dissimilarities between individual‐level and population‐level covariate effects with Wald's *χ* ^2^ test. We generated semivariograms using the package geoR (Ribeiro et al. 2024) and fit spatial mixed models using the package spaMM (Rousset and Ferdy 2014). We fit our glm and glmm models in the package lme4 (Bates et al. 2015) and performed model averaging in MuMIn (Barton 2024) using R statistical software (R Core Team 2023).

#### 2.4.3 Reproductive Class Core Habitats

We compared habitat conditions of core areas by reproductive class and ecological level using multinomial logistic regression. We fit and evaluated two models: the first compared core area habitats by reproductive class (i.e., gravid female, nongravid female, and male core habitats), and the second by ecological level (i.e., individual and population core habitats). For the two models, we specified reproductive class and level as the categorical response variable, respectively, and treated the habitat covariates as linear predictors. We interpreted the coefficients as the effects of each covariate on the log‐odds of membership in a given group relative to a chosen reference group (“gravid female” in Model 1 and “individual” in Model 2). We performed pairwise comparisons of the estimated marginal means to evaluate differences in the log‐odds of group membership based on the average values of the predictors. We interpreted significant *t*‐ratios as indications that habitat affected the likelihood of a given observation belonging to one group relative to the other. Next, we performed principal component analysis (PCA) to more easily visualize the differences in core habitats among groups. We performed Bartlett's test and examined pairwise covariate relationships to assess how well our data conformed to PCA assumptions about sphericity and linearity. Following PCA, we examined variable loadings to interpret the meanings of the group differences along the principal components (PCs) and considered loadings of |0.5| or greater to be strongly associated with a given PC. We then plotted the group means, standard errors, and 95% confidence intervals of the PC scores against the first two PCs.

To report summary statistics about the average core habitat conditions by reproductive class, and because the habitat covariates were not normally distributed, we calculated their means and standard errors using bootstrapping with 1000 iterations. We performed multinomial logistic regression using the nnet package (Venables and Ripley 2002) and pairwise significance testing using emmeans (Lenth 2023). Data manipulation and visualizations were conducted using the R packages ggplot2 and dplyr (Wickham 2024; Wickham et al. 2023).

## 3 Results

Between 2016 and 2019, we captured and radio‐tracked 25 individual copperheads 993 times over 41 snake seasons. After excluding incomplete snake seasons the dataset retained 873 telemetry relocations of 16 individual copperheads across 27 snake seasons (Table 1). Some individuals were tracked for multiple snake seasons, including several females that switched between gravid and nongravid states. Our final dataset included 5 males that contributed 9 seasons and 11 females that contributed 10 nongravid seasons and 8 gravid seasons. The average snake season consisted of 32.3 day ± 6.9 SD (range = 20–49 day) relocations (Supporting Information, Table A.2).

**TABLE 1:** Number of telemetry relocations and snake seasons by year and reproductive class after removing incomplete snake seasons.

Reproductive class	2016	2017	2018	2019
Male	0 (0)	58 (2)	118 (4)	98 (3)
Nongravid female	49 (1)	70 (2)	217 (6)	29 (1)
Gravid female	0 (0)	64 (2)	20 (1)	150 (5)

*Note:* Snake seasons are shown in parentheses.

### 3.1 Home Ranges and Space Use

Copperhead MCP‐based home range estimates, which we used to tune individual KDEs, averaged 1.2 ± 0.7 ha for gravid females, 2.2 ± 1.39 ha for nongravid females, and 5.3 ± 3.0 ha for males. Mean MCP home range estimates increased with sample size up to 20 observations and then plateaued, suggesting 20 observations was sufficient for calculation of MCP home ranges (Figure S1).

**FIGURE 2:**
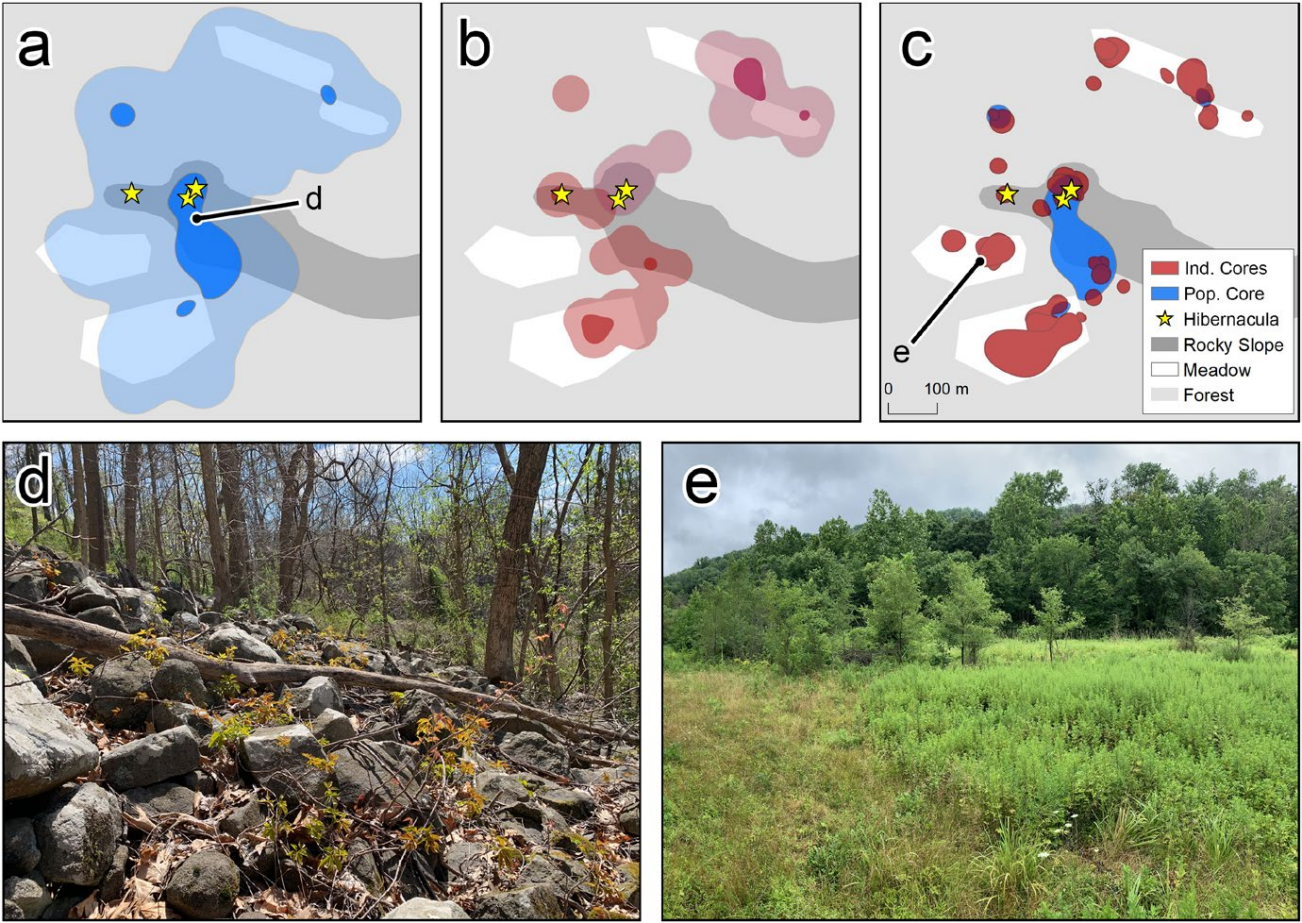
Utilization distributions derived from copperhead relocations at the population and individual levels (a—c), with dark‐shaded regions representing high‐use (i.e., core) areas and light‐shaded regions representing low use (i.e., noncore) areas. (a) UD of the entire tracked population, with core areas (dark blue) mostly coinciding spatially with rocky forested slopes surrounding hibernacula; (b) randomly selected representative snake season UDs (*n* = 2) illustrating that individual core areas (dark red) mostly coincided with summer foraging habitats; (c) juxtaposition of all individual snake season core areas (*n* = 27) and the population core area. While some individual core areas coincided with the population core area, most did not. (d, e) Representative photographs taken in a population core area (d) and an individual core area (e).

The positions of high‐use (i.e., core) and low‐use (i.e., noncore) areas depended on the level at which spatial use intensity was modeled (Figure 2). Whereas population core areas (Figure 2a) mostly coincided with communally utilized winter hibernacula and spring basking areas in rocky, forested portions of the study site, individual core areas (Figure 2b) tended to coincide with summer activity areas in edge habitats and meadows. Space use within individual copperhead home ranges was highly heterogeneous. Most home ranges contained multiple core areas, and individual and population core areas shared little spatial overlap. In general, copperheads dispersed from population core areas in May–June, remained in individual core areas from June–August, and returned to population core areas in September–October.

### 3.2 Multilevel Habitat Use

**FIGURE 3:**
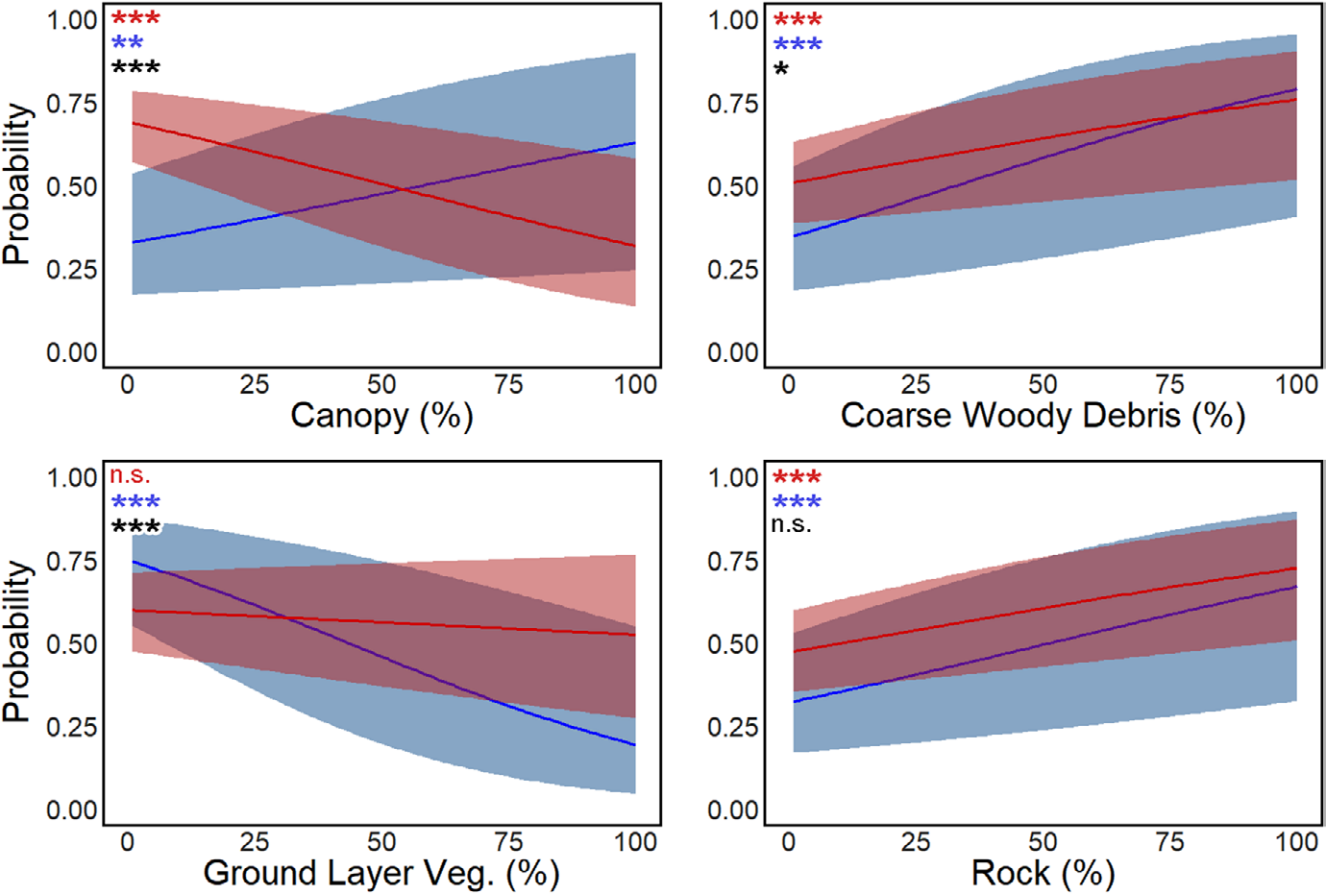
Marginal effects of selected habitat covariates on the probability that an observation originated in a core area. Curves represent best‐fit model predictions, and ribbons represent the 95% confidence intervals for individual‐level (red) and population‐level (blue) models. Asterisks indicate the significance of individual‐ and population‐level effects (red and blue, respectively) and differences between the two (black), with *** (*p* ≤ 0.001), ** (*p* ≤ 0.01), * (*p* ≤ 0.05), or *n.s*. (*p* > 0.05).

Semivariograms confirmed the presence of spatial covariance structures (i.e., spatial autocorrelation) in the habitat measurements at radio‐tracked copperhead locations (Figure S2). Copperhead spatial habitat use depended upon the ecological level at which use was modeled. For example, some habitat covariates had a weak effect on use intensity at one level but a strong effect at the other (e.g., ground layer vegetation), or had strongly opposite effects (e.g., canopy cover; Figure 3, Table 2). The effect of coarse woody debris on use intensity was positive at both individual and population levels but was significantly stronger at the population level. Individual‐level core habitats had relatively low canopy cover and woody vegetation but high amounts of rock and coarse woody debris. On the other hand, population level core habitats had high canopy, coarse woody debris, and rock cover but relatively sparse ground layer vegetation (Table 2).

**TABLE 2:** Differences in effects of (scaled) habitat covariates on copperhead use at the population and individual levels.

Estimate	*β*_ind_ (95% CI)	*β*_pop_ (95% CI)	Wald's *W*	*p*
Canopy cover*	**−0.44 (−0.6, −0.27)**	**0.44 (0.21, 0.68)**	36.74	< 0.0001
Coarse woody debris	**0.31 (0.15, 0.47)**	**0.51 (0.27, 0.74)**	1.94	0.16
Ground layer veg.*	−0.04 (−0.2, 0.12)	**−0.79 (−1.06, −0.53)**	23.07	< 0.0001
Rock cover	**0.43 (0.26, 0.61)**	**0.65 (0.41, 0.89)**	2.00	0.16
Woody veg.	**−0.16 (−0.31, −0.01)**	−0.11 (−0.34, 0.13)	0.16	0.69

*Note:* Coefficients in bold indicate significance at the *α* = 0.05 level.

* Population and individual level coefficients were significantly different (*p* < 0.05).

With respect to average habitat conditions, snake locations within population core areas had higher canopy cover (47.7% [45.6%, 49.8% CI]) than those within individual core areas (33.2% [30.8%, 35.6% CI]). While rock cover predicted core use at both the population and individual levels, it was greater in population core areas (48.1% [44.2%, 52.1% CI]) than individual core areas (36.8% [33.2%, 40.4% CI]) and was a stronger predictor of population‐level core use (*β* = 0.65 ± 0.12 SE) than individual (*β* = 0.43 ± 0.09 SE).

### 3.3 Reproductive Class Core Habitats

Core habitats differed by reproductive class (Supporting Information, Table A.3). Relative to gravid females, which were chosen as the reference group for reproductive class comparisons, there was significantly less coarse woody debris in core areas of both males (*β* = −0.98 ± 0.10 SE) and nongravid females (*β* = −0.85 ± 0.08 SE). Additionally, core habitats of nongravid females had higher canopy cover (*β* = 0.52 ± 0.09 SE), and those of males had higher ground layer vegetation (*β* = 0.40 ± 0.12 SE), than those of gravid females. Pairwise comparisons of marginal means indicated that core habitats of nongravid females differed significantly from those of both gravid females (marginal difference [MD] = −0.18 ± 0.03) and males (MD = −0.19 ± 0.03; Supporting Information, Table A.4). Although though there were significant differences in some habitat covariates between gravid female and male core areas, these differences did not translate into significant pairwise differences in marginal means (MD = 0.002 ± 0.02).

**FIGURE 4:**
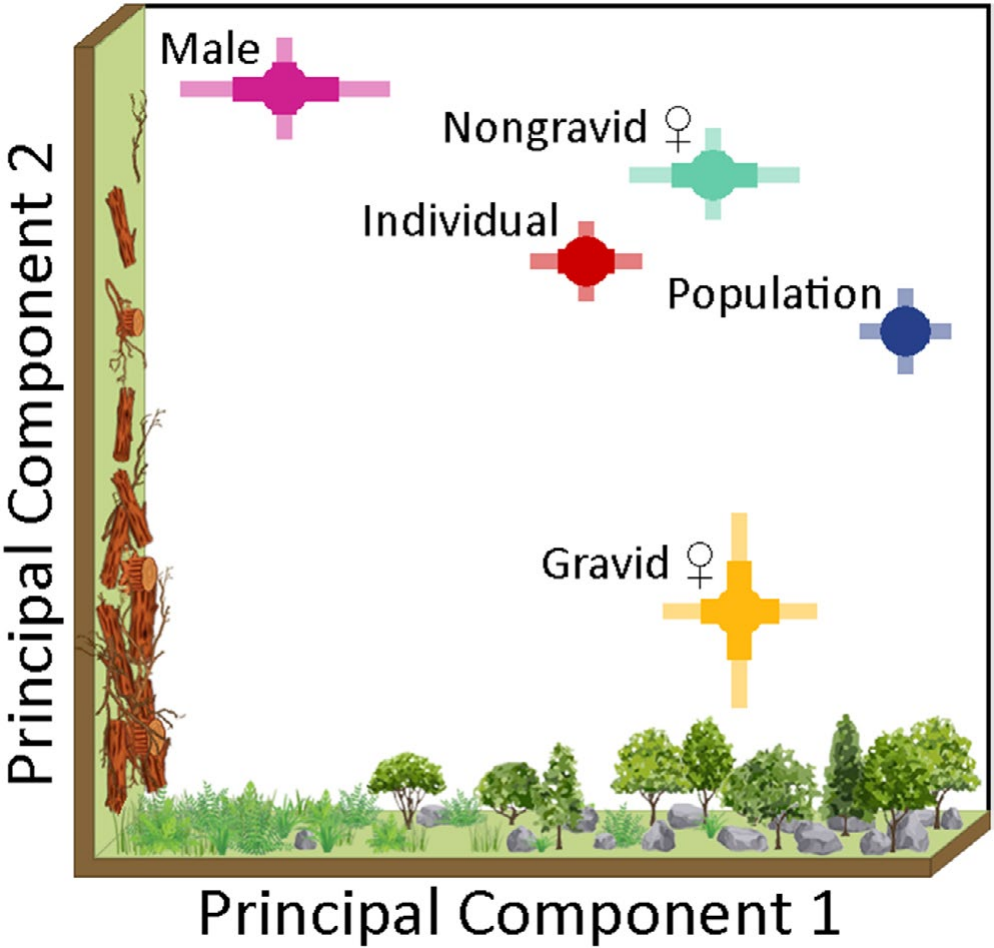
Summary results of the principal component (PC) analysis showing variation in core area habitats of gravid female, nongravid female, and male copperheads. Circles are mean PC scores, thick lines are standard errors, and thin lines are 95% confidence intervals. Axes depict only habitat covariates with strong correlations (*r* > 0.40) to the first two principal components. PC1 (a) explained 38.1% of the observed variance and was most correlated with canopy, rock, and ground layer vegetation. PC2 (b) explained 24.2% of the remaining variance and was most correlated with coarse woody debris.

Bartlett's test showed that our data were suitably nonspherical for PCA (*χ* ^2^ = 544.4, *p* < 0.0001), but there was evidence of nonlinearity among some habitat covariates (Figure S3). PCA assumes linearity among covariates, limiting its ability to accurately capture nonlinear relationships. Despite this, the first two PCs explained 62.3% of the variance among habitat covariates, indicating that the majority of the observed habitat variation within core areas was captured by these two components (Supporting Information, Table A.5). PC1 was most strongly correlated (*r* > 0.40) with canopy and rock cover (both positive), and ground layer vegetation (negative). Male core habitats had higher PC1 scores than those of gravid females and nongravid females, suggesting male core habitats had comparatively dense ground layer vegetation and low canopy and rock cover (Figure 4). PC2, which explained 24.2% of the variance among core area observations, was strongly correlated with coarse woody debris. Gravid female core areas had lower PC2 scores than those of males and nongravid females, suggesting their core area habitats contained relatively higher amounts of coarse woody debris. The low average PC1 scores of population‐level core habitats suggested they occurred in heavily forested habitats with dense rock cover and sparse ground layer vegetation.

## 4 Discussion

We found that copperhead spatial responses to habitat features differed when viewed from the individual and population levels, with individual core areas being spatially and ecologically distinct from those of the population. Both levels characterized habitats used for crucial life history functions, including sites used for communal overwintering and for individual foraging and reproduction. This suggests that investigating multiple habitat response levels, including contrasting individual and population levels, better characterized the multiple and complex interactions between copperheads and their habitats.

Home ranges of copperheads within the Sourlands were smaller than those reported elsewhere (Smith et al. 2009; Sutton et al. 2017), except for an urban copperhead population in Tennessee, USA (Flaherty 2016). All individual home ranges included the steep, rocky slopes containing hibernacula, and most included other areas of forest, canopy gaps, and human‐maintained meadows and clearings. Reptile home range size often varies due to local climatic and landscape factors, which influence how far individuals must travel to access preferred conditions (Gardiner et al. 2013; Zappalorti, Burger, and Peterson 2015; Robson and Blouin‐demers 2021). The small home ranges of Sourlands copperheads may be due to the relative proximity of suitable basking, foraging, and reproductive habitats to hibernacula.

Home range sizes in our study were smallest for gravid females, intermediate for nongravid females, and largest for males. This finding was largely consistent with reports of male copperheads having larger home ranges than both nongravid females (Smith et al. 2009) and gravid females (Sutton et al. 2017), and nongravid females having larger home ranges than gravid females (Gross 2017). In Tennessee, USA, gravid and nongravid female home ranges did not differ significantly, and only certain estimators indicated differences in home range size between females and males (Carrasco‐Harris, Cole, and Reichling 2020). Similar relative differences in home range size according to reproductive class have been observed in other snake species (Carfagno and Weatherhead 2006; Hyslop et al. 2014; Bauder et al. 2016). Male home ranges may be larger due to their larger body size or mate‐seeking movements (Smith et al. 2009; Glaudas et al. 2020; Todd and Nowakowski 2021). In contrast, gravid female ovoviviparous snakes often show reduced movement, likely a consequence of physiological trade‐offs associated with reproduction (Reinert 1984; Van Dyke and Beaupre 2011), resulting in smaller home ranges (Charland and Gregory 1995; Greene et al. 2002; Smith et al. 2009, but see Schuett et al. 2013).

Habitat features had level‐dependent influences on individual and population use, including effects that were opposite, strong at only one level, or similar across levels. For example, canopy cover had a negative effect on individual level use, but a positive effect on population level use. Other habitat features had strong effects on use at one level but weak effects on the other: ground layer vegetation, such as graminoids, herbaceous plants, and low woody vegetation, did not have a significant effect on individual use, but had a negative effect at the population level. Both rock and coarse woody debris had a significant positive effect on use at both individual and population levels. While rock was a strong predictor of use intensity at both levels, coarse woody debris was a better predictor of population‐level use intensity than it was for individuals.

Population‐level models effectively described communally used habitats, such as winter hibernacula, spring basking areas, and gestation sites, where the movements of many individuals overlapped. Hibernacula and basking areas occurred in forested, rocky portions of the study site. While use of rocky sites for hibernation aligns with reports from other northern or mountainous parts of the species' range (Fitch 1960; Fitch and Clarke 2002; Smith et al. 2009), in those reports hibernacula mostly occurred in open habitats, such as large talus slopes and outcrops, rather than closed‐canopy forest. Many snakes at northern latitudes, including copperheads, avoid dense forest during their active seasons because the darker conditions limit opportunities for thermoregulation (Reinert 1984; Smith et al. 2009, Robson and Blouin‐demers 2021). We observed copperheads basking at forested hibernacula only before trees were fully leafed out, and afterwards they quickly migrated to more open habitats. The negative effect of ground layer vegetation on population‐level use is consistent with the relatively open understory present in the rocky overwintering and spring basking sites.

Individual model inferences were generally consistent with copperheads' summer foraging and reproductive habitats. Individual core areas tended to be in meadows and edge habitats, aligning with the edge‐ and open‐habitat affinities previously documented for copperheads (Reinert 1984; Smith et al. 2009) and other north‐temperate forest snakes (Blouin‐Demers and Weatherhead 2001; Row and Blouin‐Demers 2006). Stumps, logs, and branches are consistently recognized as important features used by snakes for thermoregulation and refuge from predators (Whiles and Grubaugh 1996; Row and Blouin‐Demers 2006). Additionally, in the Sourlands, gravid female copperheads gestate in the root and soil mounds created by fallen trees (Christensen, Kwait, and Maslo 2022). This gestation behavior resulted in high amounts of coarse woody debris in gravid female core habitats and likely strengthened the estimated effect of woody debris on overall individual use. While coarse woody debris predicted both individual and population use, its effect on individual use was significantly weaker. This finding was likely because gravid females showed the strongest preference for coarse woody debris, and their communal gestation resulted in high population‐level use intensity in habitats where woody debris was ample.

One interesting result was that ground cover did not have a significant effect on individual use. Yet, we frequently observed copperheads using dense vegetation, which can aid concealment, thermoregulation, and ambushing prey (Novak et al. 2020). This suggests that individuals used sites with similar amounts of ground cover throughout much of their home ranges, demonstrating the need for careful interpretation of resource utilization parameters. Furthermore, it suggests that the open‐understory communal habitats were used briefly enough that individual models failed to represent some of their major characteristics (i.e., the scenario depicted in Figure 1b). This provides a good illustration of how individual‐based habitat models, even those that investigate multiple spatial scales or individual levels of selection, can overlook conditions of habitats with population‐level importance.

Past studies have shown that copperheads select different habitats depending on their sex and reproductive status, presumably resulting from sex‐ and state‐specific physiological demands and fitness trade‐offs (Reinert 1984; Sutton et al. 2017). While we anticipated that the differences in selection among reproductive classes would be reflected in comparisons of their core habitats, this had not been previously demonstrated. The covariate‐level comparisons and PC variable loadings indicated that male core habitats had higher ground layer vegetation and less coarse woody debris than those of gravid females. The PC loadings further suggested that male core habitats had lower canopy cover and rock cover. These inferences aligned with most males' frequent and prolonged use of open meadows from late spring through late summer. The core habitats of gravid females, on the other hand, corresponded closely with summer gestation sites. These habitats were composed of greater amounts of coarse woody debris than those of both males and nongravid females. Selection of sites with greater aboveground structure likely reflects the prioritization of thermoregulation and predator avoidance seen in many gravid female ovoviviparous snakes (Herr et al. 2020). Average PC scores and covariate means of nongravid female core habitats showed they were mostly intermediate between those of males and gravid females. The observed separation indicates that individual core habitats, not just average habitat selection throughout the home range (i.e., Reinert 1984; Sutton et al. 2017), differ for males, nongravid females, and gravid females.

While habitat separation of gravid females may be attributable to ecological trade‐offs related to ovoviviparity (Reinert 1984; Van Dyke and Beaupre 2011), male and nongravid female core habitat differences are less easily explained. We speculate that the sexual size dimorphism exhibited by copperheads may lead to sex‐dependent foraging strategies and habitat use (Weatherhead and Charland 1985; Schuett 1997; Wasko and Sasa 2012; Perkins, Cloyed, and Eason 2020). Alternatively, the greater vagility of males might have given them access to more distant summer habitats not easily accessed by nongravid females. The apparent differences in core habitats among reproductive classes may have reflected an inadequate sample of individuals rather than a positive detection of biological differences. We acknowledge this possibility while noting that the model inferences align with previous reports of differences in copperhead spatial behavior and habitat selection by sex and reproductive state (Smith et al. 2009; Reinert 1984). Regardless, our results suggest the variation in habitat use that appeared among reproductive classes was caused primarily by differences in core habitats and that the degree of niche differentiation was greatest in summer when snakes were most dispersed.

The episodic gregariousness of copperheads that led to separation of individual and population core areas is closely linked with the temporal shifts in habitat selection and movement (Smith et al. 2009). Such shifts have been documented in many temperate snake species and are driven by changing fitness demands (e.g., thermoregulation, food acquisition, reproduction, and predator avoidance) and spatiotemporally variable resources (Harvey and Weatherhead 2006; Waldron, Lanham, and Bennett 2006; Bauder et al. 2016). Because suitable conditions for reptile brumation are expected to become scarcer and more population‐limiting at increasing latitudes, large aggregations of individuals often form in winter where suitable conditions exist (Blouin‐Demers and Weatherhead 2001; Harvey and Weatherhead 2006; Williams, Hodges, and Bishop 2015). Aggregations of females that often form at gestation or oviposition sites are similarly thought to be caused by thermal constraints that become more severe at high latitudes (Graves and Duvall 1995; Harvey and Weatherhead 2010; Williams, Hodges, and Bishop 2015). In addition to habitat drivers, site fidelity and sociality may also contribute to seasonal gregariousness (Clark et al. 2012; Skinner and Miller 2020). Individuals not engaged in brumation or reproduction tend to disperse into the surrounding landscape, where they generally exist at lower population densities (Gardiner et al. 2013; Williams, Hodges, and Bishop 2015). Because this cyclical pattern is not unique to copperheads, we speculate that many snake species will possess multilevel movement patterns that can be leveraged to identify and characterize important habitats.

Temporal shifts in habitat selection make it challenging to specify models that can distinguish among habitats that are selected by animals for different biological processes, such as foraging, hibernating, and reproduction (Waldron, Lanham, and Bennett 2006; Patterson et al. 2009). One solution for snakes has been to investigate habitat responses within discrete and researcher‐defined behavioral periods (Harvey and Weatherhead 2006; Waldron, Lanham, and Bennett 2006). Other analytical approaches such as hidden Markov models are promising when state changes are associated with detectable shifts in animal behavior, such as altered movement (Klappstein, Thomas, and Michelot 2023). Such state‐dependent approaches can allow researchers to differentiate among multiple selection processes, representing an improvement over the more common approach of treating habitat selection as constant. Our approach succeeded in characterizing multiple distinct and ecologically important habitats without user‐ or model‐defined behavioral states. Intense space use at population, individual, and intraindividual levels was caused by different ecologically important behaviors (i.e., communal brumation and reproduction and individual foraging).

Effective conservation relies on accurate information about wildlife–habitat relationships. Most studies of wildlife–resource interactions have either focused on population‐level (e.g., Payer and Harrison 2003; Rittenhouse et al. 2008; Karimov, Kachel, and Hackländer 2018) or intraindividual‐level (McGarigal et al. 2016; Bauder et al. 2018) responses. However, recent examples demonstrate that individual‐level habitat inferences, even when obtained at multiple spatial scales and intraindividual levels, may not predict population‐level abundance or use intensity (McMahon et al. 2017; Marques et al. 2022, present study). We anticipate that similar multilevel patterns in space use may occur whenever habitats that attract intense individual use (i.e., individual core areas) are geographically or environmentally distinct from those that attract overlapping use by multiple individuals (i.e., population core areas). This is likely true of more taxa than just snakes, and could equally arise in scenarios where communal habitats are used asynchronously (e.g., wildlife travel corridors, watering holes, or foraging habitats). Our work adds to the growing list of studies demonstrating the need to consider multiple levels of habitat responses (Mayor et al. 2009; Zeller et al. 2017; Bauder et al. 2018), while illustrating one way in which multilevel responses may still be overlooked. For wildlife like copperheads with complex resource relationships, consideration of multilevel spatial habitat use may enhance our ability to recognize and provision habitats that satisfy all major life history requirements.

## Author Contributions

**Tyler C. Christensen:** conceptualization (lead), data curation (lead), formal analysis (lead), funding acquisition (equal), methodology (lead), visualization (lead), writing – original draft (lead). **Robert Kwait:** conceptualization (supporting), formal analysis (supporting), methodology (supporting), writing – review and editing (supporting). **Michael Van Clef:** funding acquisition (equal), project administration (equal), resources (equal), supervision (equal), writing – review and editing (supporting). **Brooke Maslo:** conceptualization (supporting), funding acquisition (equal), project administration (equal), resources (equal), supervision (equal), writing – review and editing (lead).

## Conflicts of Interest

The authors declare no conflicts of interest.

## Supporting information


**Figure S1.** Results of simulations testing the effect of number of observations on MCP home range area. For each sample size from 5 to 35 we randomly selected a snake season, randomly selected observations within that snake season, then calculated the 95% MCP home range area using those observations. We repeated this process for 100 iterations at each sample size. We then averaged across iterations to obtain an average MCP area for each sample size. The MCP area stabilized at 20 observations.

**Figure S2.** Semivariograms depicting the spatial autocorrelation structures of measured habitat covariates at copperhead‐selected locations.

**Figure S3.** Pairwise correlations and generalized additive model (GAM) fits for habitat covariates measured at copperhead locations. PCA assumes linearity among covariates, limiting its ability to accurately capture nonlinear relationships, such as those between rock cover and woody vegetation.

**Appendix S1.** Tables.

## Data Availability

Data can be accessed at https://github.com/tyler‐christensen/copperhead_habitat. We offset spatial reference data to obscure sensitive location information and to comply with conditions of New Jersey Fish and Wildlife Scientific Collecting Permits.
