# Supplementary figures and images for: Episodic Gregariousness Leads to Level‐Dependent Core Habitats: A Case Study in Eastern Copperheads (Agkistrodon contortrix)
Source: Ecol Evol. 2025 Jan 8;15(1):e70788. doi: 10.1002/ece3.70788 (PMC11707623; doi:10.1002/ece3.70788)

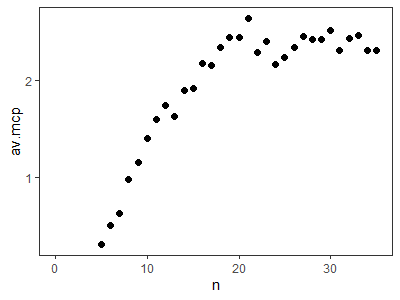

Supplement: Supplementary file 1 — Figure S1. Results of simulations testing the effect of number of observations on MCP home range area. For each sample size from 5 to 35 we randomly selected a snake season, randomly selected observations within that snake season, then calculated the 95% MCP home range area using those observations. We repeated this process for 100 iterations at each sample size. We then averaged across iterations to obtain an average MCP area for each sample size. The MCP area stabilized at 20 observations. [file ECE3-15-e70788-s001.png]

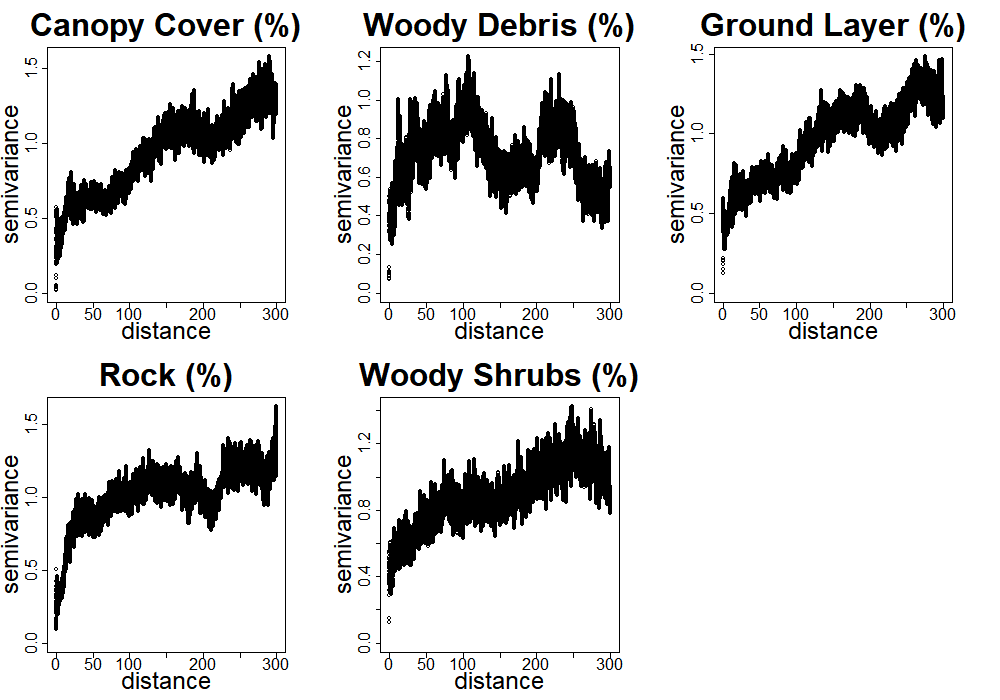

Supplement: Supplementary file 2 — Figure S2. Semivariograms depicting the spatial autocorrelation structures of measured habitat covariates at copperhead‐selected locations. [file ECE3-15-e70788-s004.png]

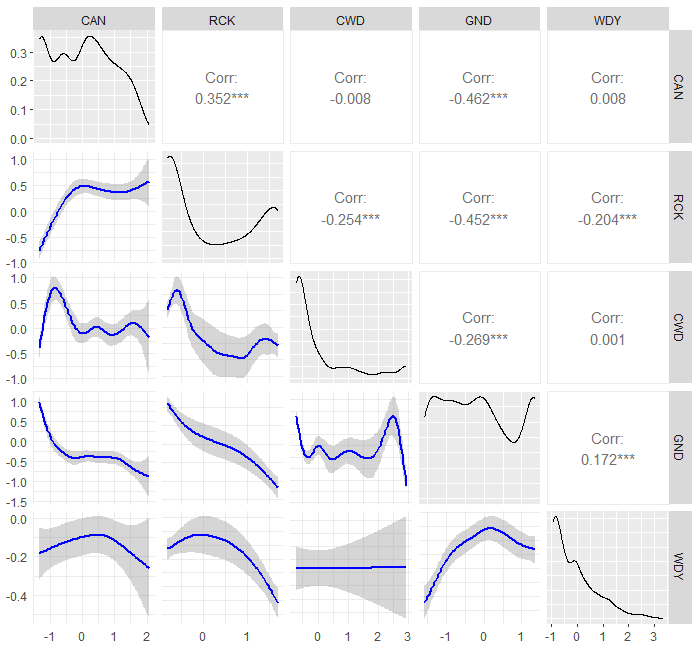

Supplement: Supplementary file 3 — Figure S3. Pairwise correlations and generalized additive model (GAM) fits for habitat covariates measured at copperhead locations. PCA assumes linearity among covariates, limiting its ability to accurately capture nonlinear relationships, such as those between rock cover and woody vegetation. [file ECE3-15-e70788-s002.png]
