# Supplementary material for: Episodic Gregariousness Leads to Level‐Dependent Core Habitats: A Case Study in Eastern Copperheads (Agkistrodon contortrix)
Source: Ecol Evol. 2025 Jan 8;15(1):e70788. doi: 10.1002/ece3.70788 (PMC11707623; doi:10.1002/ece3.70788)
Supplement: Supplementary file 4 — Appendix S1. Tables. [file ECE3-15-e70788-s003.docx]

APPENDICES

TABLES (APPENDIX)

Table A.1. Outputs of glm and glmm using a Matern spatial covariance structure. The population-level model included a random effect of snake-season. The only significant effect was canopy cover on individual level use intensity.

|  | Covariate | *β* | Cond. SE | t-value | p-value |
| --- | --- | --- | --- | --- | --- |
| Individual | Intercept | -1.8551 | 0.3588 | -5.17 | 2.911e-07 |
|  | Coarse woody debris | 0.1458 | 0.1724 | 0.8457 | 3.980e-01 |
|  | Ground layer vegetation | 0.3108 | 0.1743 | 1.7828 | 7.497e-02 |
|  | Woody vegetation | -0.2066 | 0.1457 | -1.4177 | 1.566e-01 |
|  | Rock cover | 0.3219 | 0.2014 | 1.5985 | 1.103e-01 |
|  | **Canopy cover** | **-0.622** | **0.1646** | **-3.7793** | **1.681e-04** |
| Population | Intercept | -47.6307 | 17.746 | -2.684 | 0.007413 |
|  | Coarse woody debris | 2.4567 | 2.435 | 1.0088 | 0.313347 |
|  | Ground layer vegetation | -1.0469 | 1.678 | -0.6238 | 0.532906 |
|  | Woody vegetation | 0.2703 | 1.441 | 0.1876 | 0.851265 |
|  | Rock cover | 0.3766 | 3.162 | 0.1191 | 0.905237 |
|  | Canopy cover | -0.8567 | 1.898 | -0.4514 | 0.651838 |

Table A.2. Number of telemetry relocations by individual and year. Data from years during which females were gravid are indicated with asterisks. Only data from complete snake-seasons are shown.

| Individual | Sex | 2016 | 2017 | 2018 | 2019 | Total |
| --- | --- | --- | --- | --- | --- | --- |
| A03 | ♀ | 49 | 39 | 42 | 0 | 130 |
| A05 | ♀ | 0 | 40* | 0 | 0 | 40 |
| A06 | ♂ | 0 | 32 | 38 | 30 | 100 |
| A07 | ♂ | 0 | 26 | 30 | 0 | 56 |
| A08 | ♀ | 0 | 24* | 0 | 0 | 24 |
| A09 | ♀ | 0 | 31 | 37 | 37* | 105 |
| A10 | ♀ | 0 | 0 | 44 | 0 | 44 |
| A11 | ♀ | 0 | 0 | 32 | 25* | 57 |
| A17 | ♀ | 0 | 0 | 35 | 32* | 67 |
| A18 | ♀ | 0 | 0 | 27 | 26* | 53 |
| A20 | ♀ | 0 | 0 | 20* | 0 | 20 |
| A21 | ♂ | 0 | 0 | 27 | 33 | 60 |
| A22 | ♂ | 0 | 0 | 23 | 0 | 23 |
| A27 | ♂ | 0 | 0 | 0 | 35 | 35 |
| A31 | ♀ | 0 | 0 | 0 | 30* | 30 |
| A33 | ♀ | 0 | 0 | 0 | 29 | 29 |
| Total | -- | 49 | 192 | 355 | 277 | 873 |

Table A.3. Outputs of multinomial logistic regression models comparing core habitats by reproductive class (Model 1, with gravid female as the reference group) and by ecological level (Model 2, with individual as the reference group). Coefficients represent the log-odds of membership in a given group relative to the reference group.

|  | Model 1: Reproductive Class | | | | Model 2: Ecological Level | |
| --- | --- | --- | --- | --- | --- | --- |
|  | Male | | Nongravid Female | | Population | |
| Covariate | *β* (95% CI) | *p*-value | *β* (95% CI) | *p*-value | *β* (95% CI) | *p*-value |
| Coarse Woody Debris | -0.98  (-1.18, -0.78) | < 0.0001 | -0.86  (-1.02, -0.70) | < 0.0001 | 0.16  (0.07, 0.25) | 0.003 |
| Ground Layer Veg. | 0.40  (0.16, 0.64) | 0.01 | 0.28  (0.06, 0.50) | 0.09 | -0.27  (-0.39, -0.14) | < 0.0001 |
| Woody Veg. | 0.09  (-0.13, 0.31) | 1.00 | 0.14  (-0.06, 0.34) | 1.00 | 0.15  (0.03, 0.26) | 0.06 |
| Rock Cover | -0.23  (-0.45, -0.01) | 0.35 | -0.21  (-0.39, -0.03) | 0.32 | 0.16  (0.06, 0.27) | 0.01 |
| Canopy Cover | -0.28  (-0.50, -0.06) | 0.19 | 0.53 (0.33, 0.73) | < 0.0001 | 0.36  (0.26, 0.47) | < 0.0001 |

Table A.4. Pairwise tests for differences in estimated marginal means from multinomial regression examining log-odds of group membership by habitat for reproductive class (Model 1) and ecological level (Model 2). Coefficients with significant *t*-ratios indicated that habitat affected the likelihood of an observation belonging to one group relative to the other.

| Model | Group Comparison | *β* (95% CI) | *t-*ratio | *p*-value |
| --- | --- | --- | --- | --- |
| Model 1: Reproductive Class | Gravid female – Male | 0.00 (-0.05, 0.05) | 0.118 | 1.00 |
|  | Gravid female – Nongravid female | -0.18 (-0.24, -0.13) | -6.751 | < 0.0001 |
|  | Male – Nongravid female | -0.19 (-0.24, -0.13) | -6.781 | < 0.0001 |
| Model 2: Level | Individual – Population | 0.14 (0.10, 0.18) | 6.568 | 0.0006 |

Table A.5. Principal component variable loadings and proportions of variance explained by each principal component for individual-level core habitats.

|  | PC1 | PC2 | PC3 | PC4 | PC5 |
| --- | --- | --- | --- | --- | --- |
| Canopy | -0.5275755 | -0.0396391 | -0.42004060 | 0.712394 | 0.1901394 |
| Rock | -0.5563350 | 0.3735421 | 0.00757112 | -0.526411 | 0.5232509 |
| Coarse woody debris | -0.0233322 | -0.8617319 | 0.13520656 | -0.112380 | 0.4753559 |
| Ground layer veg. | 0.5982880 | 0.3221422 | -0.01066362 | 0.272971 | 0.6809175 |
| Woody veg. | 0.2316513 | -0.1119702 | -0.89728147 | -0.358110 | -0.0210574 |
| Proportion of variance | 0.3812 | 0.2424 | 0.2018 | 0.1095 | 0.06516 |
| Cumulative proportion | 0.3812 | 0.6236 | 0.8254 | 0.9348 | 1 |
